# Local Anæsthesia

**Published:** 1884-07-20

**Authors:** A. Morgan Vance


### LOCAL ANÆSTHESIA.

By A. Morgan Vance, M.D.

I desire to briefly call your attention to the use of local anæsthesia as a substitute for general anæsthesia in many of the minor, and a few of the major operations in surgery.

So far as I can learn, the use of local anæsthesia has been very unsatisfactory in the hands of many surgeons, and has fallen into disuse. For several months I have used it in all the smaller operations with the greatest satisfaction, and believe that failures heretofore are due to the fact that the attempt has been to completely freeze the tissues, hoping to gain sufficient anæsthesia at the outset to last through the cutting operation. The difficulty being that frozen flesh is hard to cut, and about the time one gets to work the anæsthesia has departed, or is deep enough only to permit very superficial incision.

I have found ether the most suitable agent, the old method with ice and salt having the objection mentioned, that is, that one is compelled to completely freeze the part. Rhigoline is more volatile than ether, but is much more inflammable. The method of applying the ether spray is the secret of success. The atomizer with two bulbs is better than the ordinary instrument with one, as with the former the spray is constant. The assistant manipulating the spray must understand the different steps of the operation, especially if it be complicated. The spray is thrown on the part for only a moment, when the knife can be used, continuing the spray at intervals or constantly in the incisions, thus making the superficial anæsthesia precede the knife to any desired depth, no pain being felt. The ether seems to have a hemostatic effect also, as less blood is noticed than in ordinary cases. The healing process goes on as well as usual, no retardation or other bad effect being noticed in my cases. I have done tenotomies under its influence on limbs affected with infantile paralysis, in which the circulation was very poor, without sloughing or other trouble. I have done many tenotomies in adults and children, the patient experiencing absolutely no pain.

I assisted Dr. Cheatham in the removal of an eye, in case of a man of forty-five, who suffered little or no pain. Another case with the same physician, in which tracheotomy was performed upon a man of fifty, with little complaint.

At the City Hospital clinic a fibrous tumor as large as an orange was removed from a man's sacrum, the incision being six or eight inches long, with prolonged dissection on the sides of the growth. This patient did not feel the knife, but complained of the ether running down over the anus and burning severely. This could be avoided in similar cases by using oil about the mucous orifice.

In a circumcision on an adult, recently performed, no pain from the knife was felt, but the same burning was complained of about the fundament.

In tapping the abdomen, hydroceles, abscesses, or any cases in which the trocar or aspirator is used, the pain is rendered *nil* by a moment's spraying over the point. In one case, the removal of a fibrous tumor from the dorsum of the foot, the dissection was so prolonged that the ether gave out and chloroform had to be given. The cutting done under the local anæsthesia was painless, and the lady, a physician's wife, never tires of praising the spray, saying if she had a bone felon she would ride a hundred miles to have it opened under its influence.

In closing, I would ask, that in view of the dangers we know to attend the use of chloroform and ether as general anæsthetics, is it not our duty to substitute, whenever we can, a safer procedure, if thereby we can secure the same results? I am convinced that in a number of cases in which we now consider general anæsthesia absolutely necessary, the work could be, and should be done under the influence of local refrigeration. Herniotomy, ligation of vessels, amputation of fingers and toes may be performed, or, as I have shown, an eye can be removed or a tracheotomy done without difficulty.—*Louisville Med. News.*

